# Glycemic Control and Bone Turnover in Older Mexican Americans with Type 2 Diabetes

**DOI:** 10.1155/2018/7153021

**Published:** 2018-05-13

**Authors:** Nahid J. Rianon, Scott M. Smith, MinJae Lee, Hannah Pervin, Paul Musgrave, Gordon P. Watt, Shahla Nader, Sundeep Khosla, Catherine G. Ambrose, Joseph B. McCormick, Susan P. Fisher-Hoch

**Affiliations:** ^1^Department of Internal Medicine/Division of Geriatric and Palliative Medicine, UTHealth McGovern Medical School, Houston, TX, USA; ^2^Nutritional Biochemistry, Biomedical Research and Environmental Sciences Division, NASA Johnson Space Center, Houston, TX, USA; ^3^Department of Internal Medicine/Division of Clinical and Translational Science, UTHealth McGovern Medical School, Houston, TX, USA; ^4^Department of Biostatistics, The University of Texas Health Science Center at Houston (UTHealth) School of Public Health, Houston, TX, USA; ^5^Internal Medicine, Tulane Medical School, New Orleans, LA, USA; ^6^The University of Texas Health Science Center at Houston (UTHealth) School of Public Health, Brownsville, TX, USA; ^7^Department of Internal Medicine/Division of Endocrinology, UTHealth McGovern Medical School, Houston, TX, USA; ^8^Endocrinology/Internal Medicine, Mayo Clinic, Rochester, MN, USA; ^9^Mayo Clinic Center for Clinical and Translational Science, Mayo Clinic, Rochester, MN, USA; ^10^Department of Orthopaedic Surgery, UTHealth McGovern Medical School, Houston, TX, USA

## Abstract

Altered bone quality, caused by underlying metabolic changes of type 2 diabetes (T2D), has been hypothesized to cause altered bone strength and turnover leading to increased fracture risk in T2D patients. Current understanding about changes in bone turnover markers in T2D patients is mainly based on studies focused on Caucasian men and women. However, Hispanic populations have the highest prevalence of both T2D and osteoporosis in the US. We investigated associations of glycemic control (in terms of glycated hemoglobin [HbA1c]) and bone turnover rate in 69 older (≥50 years) Mexican American Cameron County Hispanic Cohort (CCHC) participants with T2D. Multivariable analyses were conducted to assess the associations between HbA1c (%), serum osteocalcin (OC), and serum sclerostin. In agreement with published reports from other racial/ethnic populations, our study found that lower bone turnover (indicated by lower serum OC) occurred in Mexican American men with T2D who had poorer glycemic control. For the women in our study, we found no significant association between glycemic control and OC. In contrast, HbA1c was positively associated with sclerostin for women, with near significance (*p* = 0.07), while no association was found in men. We recommend screening Mexican American individuals with T2D, specifically those with poor glycemic control, for bone loss and fracture risk.

## 1. Introduction

Individuals with type 2 diabetes (T2D) have 10%–30% higher risks of vertebral, hip, and other fractures than age-matched individuals without diabetes [1]. Age-related bone loss makes this risk higher in geriatric populations (≥65 years old) with T2D, and the risk remains high even after adjusting for factors contributing to falls such as sensorimotor deficiency and neuropathy [1, 2]. However, the paradox of fragility fractures occurring in the presence of normal or non-osteoporotic BMD and low bone turnover in T2D patients makes it challenging to appropriately screen these high-risk individuals for fracture risk. Currently accepted fracture screening tools, particularly the Fracture Risk Assessment Tool (FRAX), do not take T2D into account for fracture risk calculation.

Obesity with greater accumulation of visceral fat, indicating underlying metabolic changes, has been associated with low BMD [3]. The similarity of bone load-to-strength ratios in individuals with T2D and nondiabetics eliminates any benefit to BMD from greater weight due to obesity in those with T2D [3]. Nonetheless, altered bone quality, caused by underlying metabolic changes of T2D, has been hypothesized to cause altered bone strength and turnover leading to increased fracture risk in T2D patients [1, 4–7].

Few human studies have reported associations between poor glycemic control and low rates of bone turnover indicated by low serum levels of osteocalcin (OC) [8–13]. Most of the current understanding regarding changes in bone turnover markers in T2D patients has been developed from data in populations of primarily Caucasian descent. However, Hispanic populations have the highest prevalence of both T2D and osteoporosis in the USA [4]. Lack of focus on Hispanic populations and older age groups makes it difficult if not impossible to apply the results from these studies to the Hispanic minorities who are at risk of fracture due to both aging and T2D. Pronounced health disparities between Caucasians and Hispanics with higher prevalence of T2D and metabolic disorders including obesity in the latter make it critically important to learn about a better indicator of fragility fracture risk in Hispanics, America's fastest growing minority population [5, 6, 14]. The Cameron County Hispanic Cohort (CCHC) in Brownsville, Texas, follows over 1400 older (≥50 years) Mexican American men and women recruited from randomly selected households in a predominantly Mexican American community in south Texas [15, 16], the largest group of Hispanic descent with high prevalence of T2D. The CCHC thus provides a unique opportunity to investigate whether levels of glycemic control should be a concern for fracture risk due to alteration in bone turnover, a measure of bone metabolic status, in older Hispanic T2D individuals.

Low bone turnover rates in older patients are indicated by low serum OC, and patients with T2D have been shown to have low OC [10, 13]. Reduced bone formation rates have been hypothesized to be responsible for this low turnover in T2D, which may lead to increased fracture risk [12]. We hypothesize that low bone turnover associated with low levels of serum OC (caused by low formation), in the presence of high serum sclerostin, is also associated with poor glycemic control in older Hispanic patients suffering from T2D. We investigated associations of glycemic control and bone turnover rate in older (≥50 years) Mexican American CCHC participants with T2D. Specifically, we report associations of glycated hemoglobin (HbA1C), as a measure of 3-month average glycemic control, with serum levels of OC (a marker of bone turnover) and sclerostin (a regulator of bone turnover) in older Mexican Americans suffering from T2D. In addition, we determined whether duration of having T2D affects this association.

## 2. Materials and Methods

### 2.1. Population

Participants were part of the population-based older (≥50 years) cohort of men and women nested in the CCHC study. Details about the setting, data collection, and follow-up plan for the CCHC study have been published previously [15–17]. Briefly, the Cameron County Hispanic Cohort (CCHC) is a two-stage randomly selected, “Framingham-like” cohort of Mexican Americans on the USA-Mexico border. Severe health disparities exist between the population from which the CCHC was drawn and the majority of the Caucasian population. The CCHC consists of over 4000 men and women 18 years or older (1400 of whom are in the older cohort) who have been followed for more than 10 years. In a yearly survey, investigators collect information on demographics, history of fall and fracture, medication use, co-morbidities, and behavioral risk factors, such as smoking, physical activity, and alcohol use in this population. Serum samples are also collected as part of the protocol, to check for markers of various diseases, including metabolic risk factors, and for information on glucose metabolism, as from blood concentrations of HbA1c. The older cohort has been developed to study epidemiology of aging in Mexican Americans from a health disparity community who have a high prevalence of metabolic risk factors including T2D and obesity [17]. A bone health protocol was developed in 2013 to understand epidemiology of skeletal health risk factors related to age-related bone loss and fracture risk in this population. Participants receive annual DXA-acquired BMD of the whole body, hip, and spine regions. Acquisition of data on body composition and on bone turnover (serum OC level) is part of the routine bone health protocol. We report here data from 69 men (*N* = 20) and women (*N* = 49) diagnosed with T2D (according to American Diabetic Association diagnosis criteria 2010) [18].

The main CCHC study and the bone protocol were approved by the committee for protection of human subjects (CPHS) of the University of Texas Health Science Center at Houston. Participants were excluded if they had a history of active cancer, type 1 diabetes, known metabolic bone disease, or known osteoporosis; if they had a history of being treated for osteoporosis; or if they were taking medications affecting bone health, for example, thiazolidinedione. All women included in this study were postmenopausal by self-reported history of no menstrual cycle in the past 6 months.

### 2.2. Bone Densitometry

A Hologic Discovery QDR series DXA instrument was used to complete whole-body and hip scans. The left side was the default scan site, but the right side was used if the subject had a history of left hip fracture or history of surgical intervention.

### 2.3. Bone Biochemistry

Blood samples were collected using standard phlebotomy techniques during data collection visits. After clotting and centrifugation, aliquots of serum were frozen at −70°C. Samples were shipped on dry ice to the National Aeronautics and Space Administration's (NASA) Nutritional Biochemistry Laboratory at the Johnson Space Center (JSC) for subsequent analysis.

Osteocalcin levels (total) were determined by radioimmunoassay (RIA) (Alpco [Salem, NH, USA], intra- and interassay coefficients of variation [CVs] 4.9% and 6.0%, respectively, for total OC). 25-hydroxyvitamin D was determined by RIA (DiaSorin [Stillwater, MN, USA] intra- and interassay CVs 4.8% and 13.2%, respectively). Sclerostin was determined by enzyme immunoassay (Biomedica, Vienna, Austria, distributed by Alpco, intra- and interassay CVs 3.7% and 3.7%, respectively). Parathyroid hormone was determined by immunoradiometric assay (Scantibodies Laboratory, Inc. [Santee, CA, USA], intra- and interassay CVs 2.5% and 4.0%, respectively). Serum magnesium and phosphorus were determined colorimetrically (ACE Alera, Alfa Wasserman [West Caldwell, NJ, USA], intra- and interassay CVs 3.0% and 4.8% for magnesium, respectively, and 1.5% and 2.8% for phosphorus, respectively). The Nutritional Biochemistry Laboratory at JSC participates in several external quality control programs, including DEQAS and NIST.

### 2.4. HbA1c Measurements

Glycemic control was reported in terms of HbA1c levels. Blood samples collected as part of the cohort study were used for HbA1c analysis performed in the local CLIA-approved laboratory using HPLC methodology. A report showing that an HbA1c level of >8% [19] was associated with a high risk of diabetes-related complications and/or death in older adults directed consideration of HbA1c ≤8 or >8 as the dividing point for the independent variable HbA1c in our study; HbA1c >8 was considered to indicate poor glycemic control.

### 2.5. Statistical Analysis

Because of known differences in bone mineral distribution, data were analyzed separately for men and women in this cross-sectional study. Participants' characteristics were described using percentages for categorical variables and mean (±standard deviation) for continuous variables. Univariable and multivariable analyses were conducted to assess the associations between HbA1c (%) and each of the continuous outcome variables, that is, serum OC and serum sclerostin. The multivariable regression models were adjusted for age groups (dichotomized as <65 and ≥65 years [20]), BMI, femoral neck BMD, serum concentrations of creatinine (marker of renal function) skeletal health risk factors (e.g., calcium and 25-hydroxyvitamin D), and disease (T2D) duration. Interaction effects with HbA1c were evaluated while developing a final multivariable model to identify the factors that modify the associations between HbA1c and OC and between HbA1c and sclerostin. Analyses were performed using SAS 9.4 (SAS Institute Inc., Cary, NC), and alpha was set at 0.05.

## 3. Results

Table 1(a) describes general characteristics of male and female participants. Ages ranged from 51 to 84 years, with mean ± SD 66 ± 9 years for men and 67 ± 8 years for women. Average time in years since diagnosis of T2D was 12 ± 9 years for men and 13 ± 8 years for women. There were no significant differences between men and women for BMI, or for serum PTH, calcium, or 25-hydroxyvitamin D concentrations.

**Figure 1:**
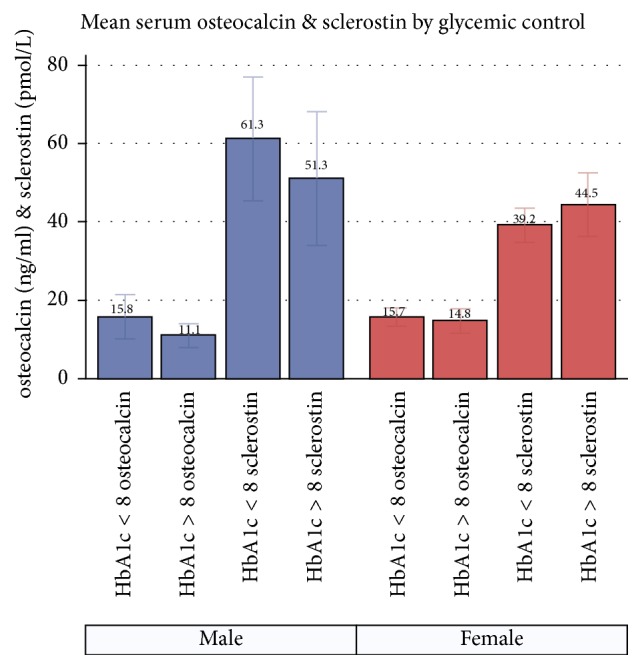
Mean distribution of serum osteocalcin and sclerostin in Mexican American men and women diagnosed with T2D, by levels of glycemic control.

Figure 1 shows mean serum OC and sclerostin by HbA1c group (i.e., poor (HbA1c > 8) or good (HbA1c ≤ 8) glycemic control) and sex. In men, mean serum OC was lower in those with poor glycemic control (HbA1c > 8%) than in those with good glycemic control (HbA1c ≤ 8%) (11.1 ± 3.9 vs 15.8 ± 8.4 ng/ml, *p* = 0.12). Mean serum sclerostin was higher in men with good glycemic control than in those with poor control (Figure 1) (61.3 ± 22.2 vs 51.3 ± 22.3 pmol/l, *p* = 0.34). While the differences in mean OC levels between women with poor and good glycemic control (14.8 ± 6.1 vs 15.7 ± 6.4 ng/ml) were not significant (*p* = 0.64), there was a trend for mean sclerostin to be higher in women with poor glycemic control than in those with good control (44.4 ± 15.8 vs 39.2 ± 11.8 pmol/l, *p* = 0.20). In men, higher mean OC and lower mean sclerostin were reported in the younger (<65 years) group than in the older group (Table 1(b)). In women, while OC was slightly higher in younger women, sclerostin levels were similar between the younger and older group.

Tables 2 and 3 show the results from the multivariable linear regression models for OC and sclerostin, respectively. Regardless of its significance, the interaction effect between HbA1c and age group was estimated for each multivariable model to show the HbA1c effect separately for younger and older age groups. A significant association between higher HbA1c (poor glycemic control) and lower OC levels was found in men in the older age group (≥65 years; *p* = 0.04) (Table 2). Longer disease duration was not significantly associated with OC in men. No significant association was found between HbA1c and OC in women. The associations between HbA1c and serum sclerostin levels were not significant in men (Table 3). In women, a positive trend of association was observed between HbA1c and sclerostin levels in both younger and older groups (*p* = 0.07 for both age groups).

## 4. Discussion

The primary goal of this study was to determine whether glycemic control in older Mexican American men and women with T2D is associated with bone turnover status as measured by serum OC and sclerostin levels. Our results show dramatic differences in these measures between men and women. We report a statistically significant negative association of glycemic control (HbA1c) with bone turnover (serum OC levels) in older (≥65 years) Mexican American men with T2D in the CCHC study. Duration of disease was not associated with serum OC (Table 2). In agreement with published reports from other racial/ethnic populations, our study found that lower bone turnover (indicated by lower serum OC) occurred in Mexican American men with T2D who had poorer glycemic control. Associations between serum sclerostin levels and glycemic control did not follow the same pattern in younger and older groups of men in our study. Poor glycemic control with higher HbA1c was positively associated with sclerostin in older men and negatively associated with sclerostin in younger men (Table 3); however, these associations were not statistically significant. For the women in our study, we found no significant association between glycemic control and OC (Table 2). However, glycemic control was positively associated with sclerostin for women in both age groups, with near significance (*p* = 0.07) (Table 3).

Mean levels of serum OC in both men and women in our study (Table 1(a)) did not reach a threshold high enough to be considered a fracture risk in an aging skeleton [21]. The lower levels of OC in older men and women (Table 1(b)) are the opposite of the known trend observed with normal aging [22, 23]. Previous studies reported associations of lower OC levels with fractures in T2D patients [11, 12]. Lower OC in men with poor glycemic control in our study (Figure 1) may indicate the altered metabolic state of the aged bone, making it more vulnerable to fracture because of underlying metabolic and structural changes. It is not completely clear why we did not see this effect in women in the current study. Although the patterns of change are similar in older men and women, women are known to have a more striking and faster increase in OC after menopause, as well as an increased rate of bone loss and higher risk of fracture relative to men of similar ages [22]. More investigation is warranted to evaluate whether bone turnover is different between the sexes because of differences in structural damage due to T2D. However, a lack of increase in OC with age in all female participants with T2D, regardless of their level of glycemic control, may be considered a metabolic change in bone for older Mexican American women. A longitudinal follow-up to assess the natural progression of disease and changes in bone turnover in older Mexican American men and women may be the next step to understand their patterns of change in bone turnover caused by T2D.

The negative associations between glycemic control and serum OC were not consistent with the high serum sclerostin (indicator of low bone formation) for both age groups and for both sexes in our study. However, we compared bone turnover with poor or good control of blood glucose levels in the older Mexican American men and women. Mean serum levels of sclerostin for men and women in our study (according to the laboratory where serum samples were analyzed) were higher than the 50th percentile of normal ranges (for men 32–67 pmol/l and for women 22–52 pmol/l). In the absence of clear normative data in clinical practice for older adults without metabolic or bone health problems, clinical judgment of high risk for fracture is often based on a trend of bone turnover that is above or below the 50th percentile. Although this study is limited by a lack of nondiabetic controls, considering the normal ranges, the higher than normal values found in our study indicate that bone formation may be low for both men and women, thereby confirming a similar pattern of bone formation in older adults with T2D reported for people from other racial/ethnic backgrounds [8, 24].

Because BMD often is misleading for fracture risk assessment in patients with T2D, adding serum OC levels to current testing, as an indicator may help to improve screening of T2D patients for their risk of fracture. Because an age-appropriate normal range for older subjects (>65 years) is unavailable, we recommend establishing a baseline level for markers of bone turnover at the time of screening with DXA BMD and continuous monitoring over time in association with HbA1c to monitor bone metabolism in the older T2D Mexican Americans.

Bone changes in women are far more complex than those in men because women undergo additional physiological changes due to estrogen deficiency, pregnancy, and lactation. Bone turnover is usually higher in women during the postmenopausal years than it is in men in the same age range [25]. While it is not clear why we did not see any association between HbA1c and OC in women in our study, a longitudinal follow-up with additional markers of resorption and formation in the same cohort may shed light on the impact of aging in these women with T2D.

This is the first study reporting relationships between glycemic control and bone turnover in an older Mexican American cohort. While our results may not be applicable to people from other racial/ethnic backgrounds, this study adds valuable scientific information about bone metabolic changes in individuals from Hispanic backgrounds who have T2D. The small number of participants, lack of nondiabetic controls, and the cross-sectional analysis of data are limitations of our study. However, none of the study participants were on medications that are known to affect bone metabolism, for example, thiazolidinedione and bisphosphonates. Future follow-up of this cohort with additional numbers of men and women with and without T2D and addition of more specific resorption and formation markers will help define the impact of T2D on fracture risk of the aging skeleton in older Mexican American men and women. The significant results in men, with a smaller sample size than women, in our study also raise concerns of worse alteration of bone metabolism in men than women, which may indicate worse risks of fragility fracture in men with T2D.

Poor glycemic control in older Mexican American men and women is considered risk of developing multiple cardio-metabolic health problems. Our study adds risk of abnormal bone metabolism to this list of concerns, and we recommend screening Mexican American individuals with T2D, specifically those with poor glycemic control, for bone loss and fracture risk.

## Figures and Tables

**Table:** (a) Participants' demographics, biochemistry, and DXA characteristics

Variables	Men (*N* = 20)	Women (*N* = 49)	*p* value
	(mean ± SD) or (*N*) %	(mean ± SD) or (*N*) %	
Age			
<65 years	(8) 40.0%	(14) 29.6%	0.40
≥65 years	(12) 60.0%	(35) 71.4%	
Height (cm)	168 ± 7.4	153 ± 6.5	<0.01
Weight (kg)	95 ± 26.7	75 ± 19.9	<0.01
BMI (kg/m^2^)	32 ± 5.8	32 ± 8.6	0.92
HbA1c (%)	8.2 ± 1.8	7.8 ± 1.5	0.35
Years since T2D was diagnosed			
<10 years	(9) 52.9%	(16) 39.0%	0.39
≥10 years	(8) 47.1%	(25) 61.0%	
Serum creatinine (mg/dl)	1.0 ± 0.2	0.8 ± 0.2	0.01
Serum calcium (mmol/l)	2.3 ± 0.1	2.4 ± 0.1	0.13
Serum 25-hydroxyvitamin D (nmol/l)	65.2 ± 23.1	59.6 ± 19.0	0.31
Serum magnesium (mmol/l)	0.7 ± 0.1	0.71 ± 0.09	0.58
Serum phosphorus (mmol/l)	1.0 ± 0.2	1.2 ± 0.2	<0.01
Serum PTH (pg/ml)	33.8 ± 15.2	27.8 ± 13.7	0.12
Femur neck BMD (g/cm^2^)	0.8 ± 0.1	0.7 ± 0.1	<0.01
Femur neck *T*-score	−1.3 ± 0.9	−1.6 ± 1.0	0.32
Serum osteocalcin (ng/ml)	13.7 ± 7.0	15.4 ± 6.3	0.33
Serum sclerostin (pmol/l)	56.5 ± 22.2	41.1 ± 13.4	<0.01

*Note*. SD: standard deviation; HbA1c: glycated hemoglobin; T2D: type 2 diabetes; PTH: parathyroid hormone. All participants were Mexican American and had been diagnosed with T2D.

**Table:** (b) Mean distribution of osteocalcin and sclerostin by age group in Mexican American men and women diagnosed with T2D

Variables	Subgroup category	Men	*p* value	Women	*p* value
		mean ± SD		mean ± SD	
Osteocalcin (ng/ml)	Age group < 65 years	15.6 ± 9	0.25	16.6 ± 6	0.37
	Age group ≥ 65 years	11.9 ± 5		14.8 ± 7	
Sclerostin (pmol/l)	Age group < 65 years	50.9 ± 18	0.32	41.1 ± 14	0.90
	Age group ≥ 65 years	60.9 ± 24		40.5 ± 13	

*Note*. SD: standard deviation.

**Table 2:** Multivariable association between osteocalcin and HbA1c for Mexican American men and women diagnosed with T2D.

Variables	Men		Women	
	Adjusted mean difference (95% CI)	*p* value	Adjusted mean difference (95% CI)	*p* value
*HbA1c*				
**Age ≥ 65 group **	**−4.93 (−9.47, −0.38)**	**0.04**	**−0.44 (−1.89, 1.01)**	**0.54**
**Age < 65 group**	**−2.78 (−6.28, 0.72)**	**0.10**	**−0.70 (−2.10, 0.70)**	**0.31**
BMI	−0.19 (−2.23, 1.85)	0.82	−0.25 (−0.68, 0.18)	0.24
Serum creatinine	34.07 (−20.20, 88.34)	0.17	12.79 (2.99, 22.60)	0.01
Serum calcium	−4.59 (−144.13, 134.95)	0.94	−35.56 (−61.90, −9.21)	0.01
Serum vitamin D	0.15 (−0.30, 0.60)	0.44	−0.08 (−0.20, 0.05)	0.24
Femur neck BMD	3.21 (−48.32, 54.74)	0.88	11.42 (−13.57, 36.40)	0.36
≥10 years since T2D diagnosis	−1.04 (−14.55, 12.47)	0.85	1.17 (−3.63, 5.96)	0.62

*Note*. CI: confidence interval; interaction effect between HbA1c and age for men *p* = 0.81, for women *p* = 0.79.

**Table 3:** Multivariable association between sclerostin and HbA1c for Mexican American men and women diagnosed with T2D.

Variables	Men		Women	
	Adjusted mean difference (95% CI)	*p* value	Adjusted mean difference (95% CI)	*p* value
*HbA1c*				
**Age ≥ 65 group **	**0.37 (−15.77, 16.50)**	**0.96**	**2.52 (−0.24, 5.28)**	**0.07**
**Age < 65 group**	**−1.80 (−14.20, 10.60)**	**0.72**	**2.45 (−0.19, 5.09)**	**0.07**
BMI	−3.26 (−10.49, 3.96)	0.30	−0.93 (−1.73, −0.13)	0.02
Serum creatinine	−73.71 (−266.19, 118.77)	0.37	29.05 (10.82, 47.29)	0.003
Serum calcium	−33.54 (−528.40, 461.31)	0.87	0.15 (−48.80, 49.09)	0.99
Serum vitamin D	−0.96 (−2.56, 0.63)	0.18	0.03 (−0.21, 0.27)	0.82
Femur neck BMD	40.48 (−142.27, 223.22)	0.59	94.31 (47.77, 140.87)	0.0003
≥10 years since T2D diagnosis	11.93 (−35.99, 59.84)	0.55	4.43 (−4.49, 13.35)	0.32

*Note*. CI: confidence interval; interaction effect between HbA1c and age for men *p* = 0.77, for women *p* = 0.29.
